# A 21-bp InDel in the promoter of *STP1* selected during tomato improvement accounts for soluble solid content in fruits

**DOI:** 10.1093/hr/uhad009

**Published:** 2023-02-10

**Authors:** Ying Wang, Chunmei Shi, Pingfei Ge, Fangman Li, Lihui Zhu, Yaru Wang, Jinbao Tao, Xingyu Zhang, Haiqiang Dong, Wenxian Gai, Fei Wang, Zhibiao Ye, Donald Grierson, Wei Xu, Yuyang Zhang

**Affiliations:** National Key Laboratory for Germplasm Innovation and Utilization of Horticultural Crops, College of Horticulture and Forestry Science, Huazhong Agricultural University, Wuhan 430070, China; National Key Laboratory for Germplasm Innovation and Utilization of Horticultural Crops, College of Horticulture and Forestry Science, Huazhong Agricultural University, Wuhan 430070, China; National Key Laboratory for Germplasm Innovation and Utilization of Horticultural Crops, College of Horticulture and Forestry Science, Huazhong Agricultural University, Wuhan 430070, China; National Key Laboratory for Germplasm Innovation and Utilization of Horticultural Crops, College of Horticulture and Forestry Science, Huazhong Agricultural University, Wuhan 430070, China; National Key Laboratory for Germplasm Innovation and Utilization of Horticultural Crops, College of Horticulture and Forestry Science, Huazhong Agricultural University, Wuhan 430070, China; National Key Laboratory for Germplasm Innovation and Utilization of Horticultural Crops, College of Horticulture and Forestry Science, Huazhong Agricultural University, Wuhan 430070, China; National Key Laboratory for Germplasm Innovation and Utilization of Horticultural Crops, College of Horticulture and Forestry Science, Huazhong Agricultural University, Wuhan 430070, China; National Key Laboratory for Germplasm Innovation and Utilization of Horticultural Crops, College of Horticulture and Forestry Science, Huazhong Agricultural University, Wuhan 430070, China; National Key Laboratory for Germplasm Innovation and Utilization of Horticultural Crops, College of Horticulture and Forestry Science, Huazhong Agricultural University, Wuhan 430070, China; National Key Laboratory for Germplasm Innovation and Utilization of Horticultural Crops, College of Horticulture and Forestry Science, Huazhong Agricultural University, Wuhan 430070, China; National Key Laboratory for Germplasm Innovation and Utilization of Horticultural Crops, College of Horticulture and Forestry Science, Huazhong Agricultural University, Wuhan 430070, China; National Key Laboratory for Germplasm Innovation and Utilization of Horticultural Crops, College of Horticulture and Forestry Science, Huazhong Agricultural University, Wuhan 430070, China; Plant Sciences Division, School of Biosciences, University of Nottingham, Sutton Bonington Campus, Loughborough LE12 5RD, UK; Key Laboratory of Special Fruits and Vegetables Cultivation Physiology and Germplasm Resources Utilization (Xinjiang Production and Construction Crops), College of Agriculture, Shihezi University, Shihezi 832003, Xinjiang, China; National Key Laboratory for Germplasm Innovation and Utilization of Horticultural Crops, College of Horticulture and Forestry Science, Huazhong Agricultural University, Wuhan 430070, China; Hubei Hongshan Laboratory, Wuhan 430070, China

## Abstract

Domestication and improvement are important processes that generate the variation in genome and phonotypes underlying crop improvement. Unfortunately, during selection for certain attributes, other valuable traits may be inadvertently discarded. One example is the decline in fruit soluble solids content (SSC) during tomato breeding. Several genetic loci for SSC have been identified, but few reports on the underlying mechanisms are available. In this study we performed a genome-wide association study (GWAS) for SSC of the red-ripe fruits in a population consisting of 481 tomato accessions with large natural variations and found a new quantitative trait locus, *STP1*, encoding a sugar transporter protein. The causal variation of *STP1*, a 21-bp InDel located in the promoter region 1124 bp upstream of the start codon, alters its expression. *STP1*^*Insertion*^ accessions with an 21-bp insertion have higher SSC than *STP1*^*Deletion*^ accessions with the 21-bp deletion. Knockout of *STP1* in TS-23 with high SSC using CRISPR/Cas9 greatly decreased SSC in fruits. *In vivo* and *in vitro* assays demonstrated that ZAT10-LIKE, a zinc finger protein transcription factor (ZFP TF), can specifically bind to the promoter of *STP1*^*Insertion*^ to enhance *STP1* expression, but not to the promoter of *STP1*^*Deletion*^, leading to lower fruit SSC in modern tomatoes. Diversity analysis revealed that *STP1* was selected during tomato improvement. Taking these results together, we identified a naturally occurring causal variation underlying SSC in tomato, and a new role for ZFP TFs in regulating sugar transporters. The findings enrich our understanding of tomato evolution and domestication, and provide a genetic basis for genome design for improving fruit taste.

## Introduction

Tomato (*Solanum lycopersicum*) is one of the most widely cultivated vegetables worldwide [1] and tomato fruit is an vital source of nutrition in the human diet [2]. The content of flavor chemicals, including volatiles, acids, and sugars largely determines consumer liking in tomato fruits [3]. The soluble solids content (SSC) is a comprehensive index to characterize soluble sugars and organic acids that make an important contribution to taste and flavor perception.

The content of soluble sugars and organic acids is a quantitative trait controlled by multiple genes. Thus far, several loci have been found to regulate accumulation of soluble sugars in tomato. The sucrose accumulator gene (*sucr*) from *Solanum chmielewskii* can confer enhanced fruit sugar composition [4]. Brix9-2-5 increased sugar yield in tomato and further analysis showed that *LIN5*, encoding a flower- and fruit-specific invertase, was mapped within this region. Variation of an amino acid led to a decrease in its enzyme activity [5, 6]. RNAi lines of *LIN5* significantly reduced the Brix content in tomato fruits [7]. Introgression of the *Fgr^H^* allele from the wild species (LA1777) into cultivated tomato reduced glucose levels and increased fructose levels and thus the fructose-to-glucose ratio of the red-ripe fruits was increased. Further research showed that the *SlFgr* gene is a member of the SWEET family, encoding plasma membrane-localized glucose efflux transporters [8, 9]. The above quantitative trait loci (QTLs) were characterized based on recombinant inbred lines (RILs) or near-isogenic lines (NILs). Tomato genome sequencing and comprehensive evolution analysis have provided molecular insights into gene function during evolution [10, 11].

Genome-wide association study (GWAS) is a powerful technology to deepen insights into the evolution of agronomically important traits and provides loci for genetic improvement [12]. Metabolic genome-wide association study (mGWAS) is one of the strongest tools for genetic determinants for differential accumulation of metabolites and diversity of plant metabolism. mGWAS was applied in *Arabidopsis thaliana* initially and subsequently extended with improvements to other species, especially important crops [13], providing deeper insights into the genetic bases of metabolic diversity [14–20]. In tomato, several genes related to SSC have been mapped through mGWAS and their natural variations have been analyzed. Typically, a 3-bp InDel in the promoter of *SlALMT9* was related to high malate content in fruits [21]. This InDel prevented SlWRKY42 from binding the promoter of *SlALMT9*, which alleviated the expression of *SlALMT9* and promoted the accumulation of high fruit malate [21]. An 8-bp InDel in the promoter of *SlbHLH59* directly regulated the expression of genes related to ascorbate biosynthesis and thereby determined ascorbate content in tomato fruits [22].

In tomato fruits, soluble sugars mainly comprise glucose and fructose, in near-equimolar quantities [9, 23], which play vital roles as sources of energy and as primary metabolites [24]. Uptake of glucose and fructose is mediated by sugar transport proteins (STPs) [25]. Originally, the three *STP* genes *LeHT1*/*2*/*3* were identified from the same family [26]. Subsequently the STP family was classified as the largest subfamily of sugar transporters in tomato [24]. RNAi lines of *LeHT* genes led to a 55% decrease in hexose accumulation in fruits [27]. Heterologous expression of *LeHT1* in yeast can rescue a hexose transport-impaired mutant and *LeHT1* showed the same transport characteristics as the high-affinity glucose/H^+^ cotransporter [27]. Despite the functional identification of sugar transporters in plants, the causal variation and regulatory mechanism remain largely unknown.

Many biological processes are regulated at the transcriptional level, including responses to the environment, balance in metabolism and physiology, and progression through the cell cycle [28]. Zinc finger protein transcription factors (ZFP TFs) usually show sequence-specific DNA binding and can activate and/or repress gene transcription [28]. ZFP TFs participated widely in stress responses and plant development [29, 30]. Overexpression of *SlCZFP1* enhanced tolerance to cold treatments or freezing in transgenic *Arabidopsis* and rice [31]. Overexpression of *ZFP179* increased salt tolerance in rice [32]. AtZAT10, a C_2_H_2_-EAR zinc finger protein, can elevate the expression of genes related to reactive oxygen defense and enhance the tolerance of osmotic stress, heat, and salinity in *Arabidopsis* plants [33]. MdZAT10 markedly accelerated leaf senescence and elevated the expression of genes related to senescence in apple [34]. In *Arabidopsis*, a few zinc finger proteins have been identified to participate in the regulation of the flowering time of *Arabidopsis*, such as JAGGED (JAD) [35] and SUPPRESSOR OF FRIGIDA4 (SUF4) [36]. The B-box (BBX) proteins, a class of ZFP TFs, are indispensable factors in controlling plant growth and development [37–40]. However, there is still a big gap between the functions of ZFP TFs in regulating sugar transporters that needs to be filled.

In this study, we measured the SSC of red-ripe fruits in 481 tomato accessions. Through mGWAS, we identified reliable QTLs that contribute to fruit SSC. Furthermore, we determined a new QTL, *STP1* (*Sugar Transport Protein 1*) by analyzing gene expression, genetic variations, and functional verification, and confirmed its contribution to high SSC in tomato fruits. In addition, we found that ZAT10-LIKE, a ZFP TF, cannot bind to the promoter of *STP1* with a 21-bp deletion, but specifically binds to the promoter of *STP1* with a 21-bp insertion, and positively regulates gene expression of *STP1^Insertion^*. These findings provide genetic insights into the evolution and regulation of SSC and gene resources for fruit quality improvement.

## Results

### GWAS reveals a locus, *STP1*, influencing tomato SSC in chromosome 2

**Figure 1:**
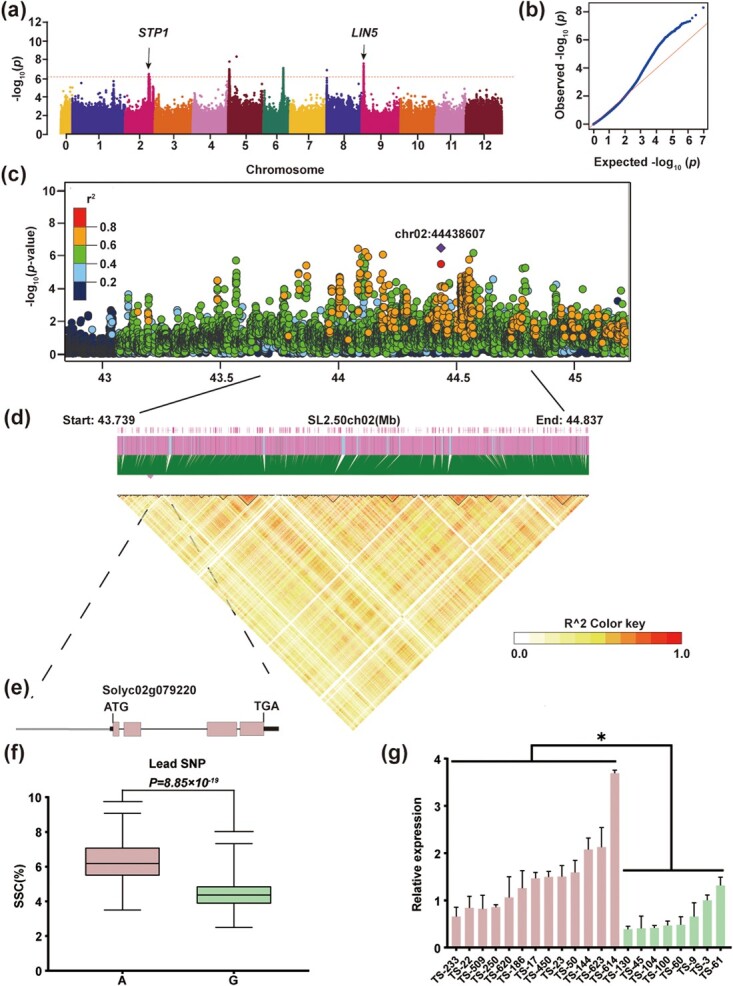
GWAS of SSC in tomato fruits. **a** Manhattan plot for GWAS result of fruit SSC (*n* = 481). The *y*-axis represents observed *P*-values transformed from minus log_10_. The genome-wide suggestive threshold (*P* = 9.93 × 10^−7^) is indicated by the red horizontal dashed line. **b** Quantile–quantile plot of expected versus observed *P*-values for GWAS. **c** Genome-wide association signals for SSC are shown in the region of 42.8–45.2 Mb on chromosome 2 (*x*-axis). The purple dot indicates the lead SNP and the color of each plot corresponds to the *r^2^* value (a measure of linkage disequilibrium (LD)) according to the legend. **d** Representation of pairwise *r^2^* values (a measure of LD) among all polymorphic sites in the 1.1-Mb genomic region corresponding to (**c**). **e** Gene structure of *STP1* (*Solyc02g079220*). The pink box and thin black lines represent coding sequence and introns, respectively. The thick black lines represent the 5′-untranslated (5′-UTR) and 3′-untranslated (3′-UTR) regions. The gray line represents the promoter. **f** Box diagram of SSC in two sets of genotypes distinguished based on the lead SNP, Chr02:44438607. **g** Relative expression of *STP1* in red-ripe fruits of different accessions. Data represent means ± standard deviation (*n* = 3). The asterisk indicates a significant difference: ^*^P ≤ .05.

Tomato SSC is a complicated quantitative trait controlled by multiple genes. Previous studies have found several QTLs that have a significant influence on SSC in tomato. In order to discover new QTLs, we determined SSC of red-ripe fruits in 481 accessions, performed a GWAS (Supplementary Data Table S1), and identified a single-nucleotide polymorphism (SNP) (SL2.50ch02:44438607) that showed a strong correlation with the level of SSC (*P* = 3.45 × 10^−7^) (Fig. 1a and b). Two alleles based on the lead SNP (SL2.50ch02:44438607, A/G) from the association signal—high-SSC allele (A) and low-SSC allele (G),were associated with high-SSC and low-SSC phenotypes in tomato, respectively (Fig. 1f). We examined the DNA sequences within 2 Mb of the lead SNP (SL2.50ch02:44438607), and 39 genes were identified (Fig. 1c and d, Supplementary Data Table S3). The gene annotated as *Sugar Transporter Protein 1*, *Solyc02g079220*, was selected and named *STP1* (Fig. 1e). To identify whether *STP1* has a major regulatory role for SSC, 13 accessions with high SSC and 8 accessions with low SSC were selected to quantify *STP1* expression in red-ripe fruits using quantitative real-time PCR (qRT–PCR). The expression levels of *STP1* were markedly higher in the high-SSC accessions than low-SSC accessions (Fig. 1g), which indicates that *STP1* is a major candidate for having an important influence on SSC in tomato.

### A 21-bp InDel in the promoter of *STP1* alters its expression

**Figure 2:**
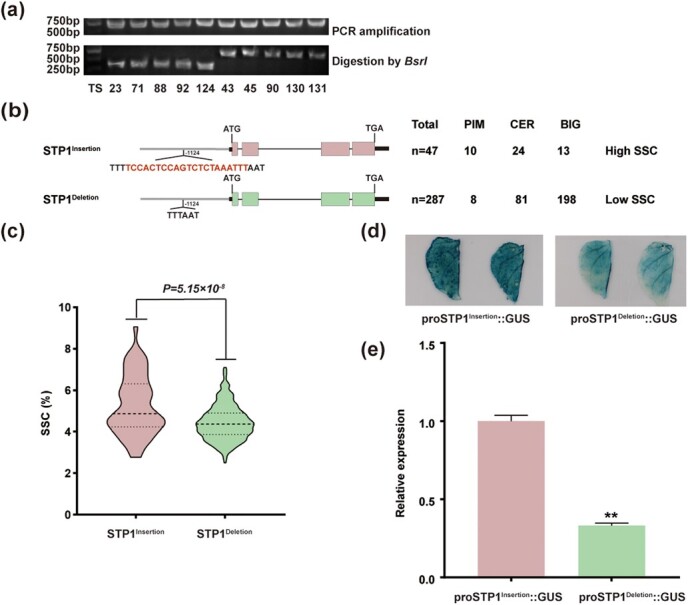
Structural variations and activity of the *STP1* promoters in high-SSC and low-SSC alleles among tomato accessions. **a** CAPS marker after digestion by *Bsr*I shows a 21-bp InDel variation in the *STP1* promoter (partial results). Upper part is the result of PCR amplification and the lower part shows the result of digestion by *Bsr*I. Bands around 750 bp represent accessions with *STP1^Deletion^* and bands around 380 bp represent accessions with *STP1^Insertion^*. **b** Structural variations of *STP1* in 334 tomato accessions. Gene structure is as in Fig. 1e. The 21-bp InDel is marked in red. PIM, CER, and BIG represent subgroups covering two *STP1* alleles (*STP1^Insertion^* and *STP1^Deletion^*). The numbers below Total, PIM, CER, and BIG are numbers of accessions. **c** SSC in red-ripe fruits of two *STP1* alleles. **d** GUS staining of tobacco leaves infiltrated with *Agrobacterium* harboring proSTP1^Insertion^::GUS and proSTP1^Deletion^::GUS, respectively. The promoters of *STP1^Insertion^* and *STP1^Deletion^* were inserted into the GUS expression vector driving the *GUS* gene and were infiltrated into tobacco leaves. **e***GUS* expression shown in (**d**) was evaluated. Asterisks indicate a significant difference: ^**^P ≤ .01.

To determine the sequences variation in *STP1*, the genomic regions surrounding *STP1* including the introns, exons, promoters, 5′-UTR and 3′-UTR were sequenced in 10 accessions with high SSC and 10 accessions with low SSC. At 1124 bp upstream of the start codon, we found a 21-bp InDel. We further checked the presence and absence of the 21-bp InDel among 334 tomato accessions by PCR amplification and restriction enzyme digestion (Fig. 2a, Supplementary Data Table S4). The test results indicated that there was a 21-bp insertion in 47 accessions and a 21-bp deletion in 287 accessions (Fig. 2b). The 21-bp insertion occurred mainly in *Solanum pimpinellifolium* (PIM) and *Solanum lycopersicum* var. *cerasiforme* (CER), and the 21-bp deletion occurred mainly in *S. lycopersicum* (BIG). This suggests that *STP1* may have been selected during tomato improvement.

By examining the sequencing results and significant SNPs within *STP1*, two different alleles were identified and named *STP1^Insertion^* and *STP1^Deletion^* (Fig. 2b). We wondered if the SSC variation between the two alleles was attributable to the 21-bp InDel in the promoter, so the frequency distribution of *STP1^Insertion^* and *STP1^Deletion^* was analyzed (Supplementary Data Table S4). Interestingly, *STP1^Insertion^* accessions contained significantly higher SSC (5.25) than *STP1^Deletion^* accessions (4.43) (Fig. 2c).

To further illustrate that 21-bp InDel on the *STP1* promoter can indeed alter its activation capacity, β-glucuronidase (GUS) staining assays were carried out. Two GUS reporter vectors, proSTP1^Insertion^::GUS and proSTP1^Deletion^::GUS, were constructed, and each was transiently expressed in tobacco leaves. Leaves infiltrated with proSTP1^Insertion^::GUS showed darker GUS staining than those infiltrated with proSTP1^Deletion^::GUS (Fig. 2d). We further quantified *GUS* expression and found that *GUS* expression driven by proSTP1^Insertion^ was approximately twice that of *GUS* expression driven by proSTP1^Deletion^ (Fig. 2e). These data confirm that possession of the 21-bp insertion enhanced the promoter activity of *STP1*.

### *STP1* mutation decreased SSC in tomato

**Figure 3:**
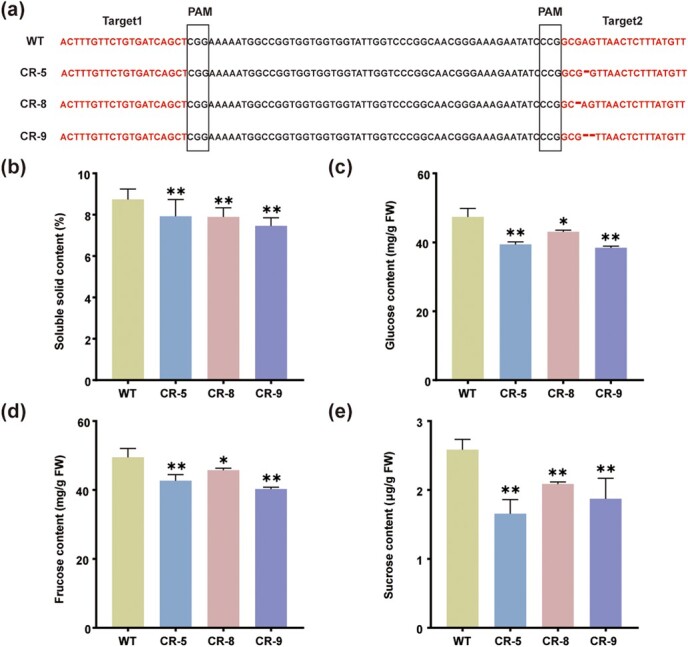
SSC, glucose, fructose, and sucrose levels in *STP1* knockout lines and WT. **a** Different types of editing in *STP1* knockout lines. WT represents background accession TS-23 with high SSC. CR-5, CR-8, and CR-9 represent knockout lines with deletions of A, G, and AG in the coding region of *STP1*, respectively. **b**–**e** Determination of SSC (**b**), glucose (**c**), fructose (**d**), and sucrose (**e**) in red-ripe fruits of three knockout lines and WT. Data represent means ± standard deviation (*n* = 3). Asterisks indicate significant differences: ^*^P ≤ .05, ^**^P ≤ .01.

To further study the role of *STP1* in SSC of tomato fruits, CRISPR/Cas9 technology was used to generate knockout lines in the TS-23 background with high SSC. Through PCR amplification and sequencing, we obtained three homozygous lines with different types of editing, CR-5, CR-8, and CR-9. CR-5 and CR-8 showed a 1-bp deletion and CR-9 harbored a 2-bp deletion in the second target. The deletions in the three lines all caused frameshift mutations in the coding region of *STP1*, which would be expected to disrupt the STP1 protein structure and transport activity (Fig. 3a, Supplementary Data Fig. S1a). We also found that *STP1* expression was suppressed in three *STP1* knockout lines using qRT–PCR (Supplementary Data Fig. S1b). We performed SSC phenotyping of three knockout lines and wild-type (WT), and the results showed that SSC decreased significantly in three knockout lines (Fig. 3b). Previous studies have shown that SlSTP1, an STP, exhibited glucose and fructose transport activity in yeast [27]. SSC in tomato mainly comprises three soluble sugars (glucose, fructose, and sucrose) and two organic acids (malic acid and citric acid). We speculated that *STP1* knockout lines may alter SSC by decreasing sugar content. We further determined the contents of glucose, fructose, sucrose, malic acid, and citric acid in red-ripe fruits of three knockout lines and WT. As expected, the contents of glucose, fructose, and sucrose significantly decreased (Fig. 3c–e), while the contents of malic acid and citric acid were unaffected and remained similar to the control (Supplementary Data Fig. S2). These data indicate that *STP1* positively affects the SSC by regulating the contents of glucose, fructose, and sucrose in tomato fruits.

### ZAT10-LIKE, a zinc finger protein transcription factor, positively regulates gene expression of *STP1^Insertion^*

In order to study how the 21-bp InDel in the *STP1* promoter regulates the expression of *STP1*, we carried out yeast one-hybrid screening. We took the 21-bp InDel sequence in the *STP1* promoter as the center and added 9-bp on the left and right sides, yielding proSTP1^Insertion^ and proSTP1^Deletion^ (Supplementary Data Fig. S3). Putatively *STP1* promoter binding proteins were retrieved and sequenced (Supplementary Data Table S5).

**Figure 4:**
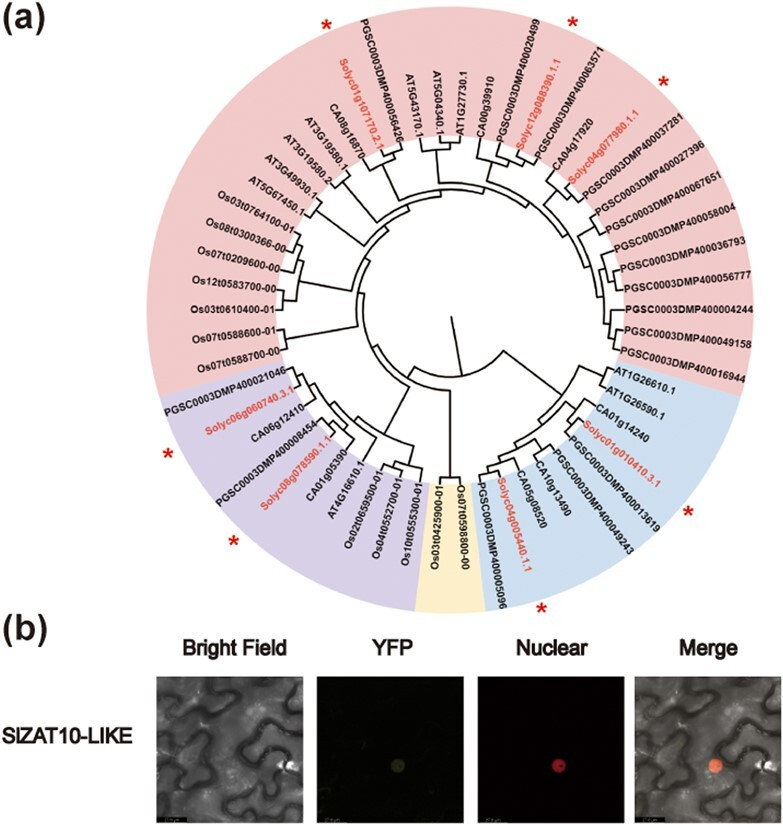
Molecular characterization of ZAT10-LIKE. **a** Phylogenetic tree of ZAT10-LIKE with its homologs from other plant species. Multiple sequences were aligned with the amino acid sequence of tomato ZAT10-LIKE and its homologs from *Arabidopsis * (**Arabidopsis *thaliana*), rice (*Oryza sativa*), pepper (*Capsicum annuum*), and potato (*Solanum tuberosum*). The asterisks indicate ZAT10-LIKE (Solyc12g088390) and its homologs in tomato. **b** Subcellular localization of ZAT10-LIKE-YFP fusion protein. The fusion construct (ZAT10-LIKE-YFP) and a nuclear marker (StERF3-RFP) were transiently expressed in tobacco leaves. Bright-field, yellow fluorescent, red fluorescent, and merged images are shown.

Two frequently occurring genes, *Solyc12g088390* and *Solyc04g077980*, were retrieved as candidate genes (Supplementary Data Table S5). Evolutionary analysis showed that these two genes are homologous genes in tomato with a homology of 52% (Fig. 4a), and were named *ZAT10-LIKE* and *ZAT10*, respectively. Subcellular localization results showed that ZAT10-LIKE and ZAT10 were both located in the nucleus (Fig. 4b; Supplementary Data Fig. S4).

**Figure 5:**
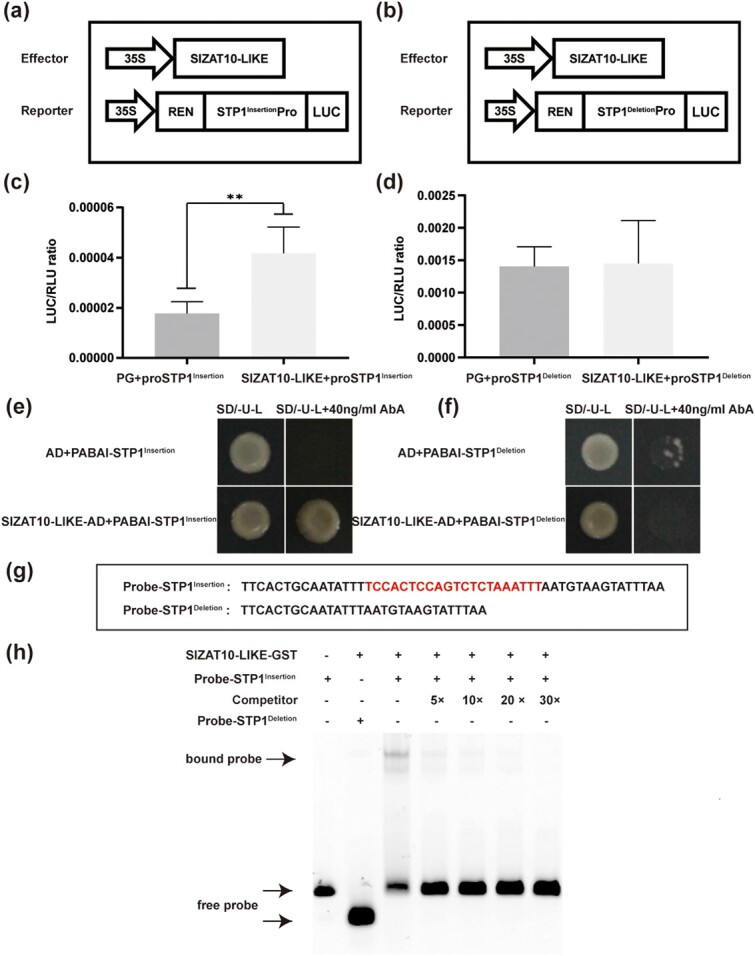
Differential binding capacity of ZAT10-LIKE to the promoters of *STP1^Insertion^* and *STP1^Deletion^*. **a**, **b** Schematic representation of constructs for the dual luciferase assays. The full-length CDS of *ZAT10-LIKE* was cloned into pGreen II 62-SK to generate an effector construct, pGreen II 62-SK-ZAT10-LIKE. The promoter fragments of *STP1* (−1 to −1187), amplified from TS-23 and TS-9, respectively, were cloned into pGreen II 0800-LUC to create the reporter constructs pGreen II 0800-STP1^Insertion^-Pro (**a**) and pGreen II 0800-STP1^Deletion^-Pro (**b**). **c**, **d** Relative LUC/REN ratios were used to evaluate the promoter activity of *STP1^Insertion^* (**c**) and *STP1^Deletion^* (**d**) in the presence of ZAT10-LIKE. PG, the pGreenII 62-SK empty vector with pGreen II 0800-STP1^Insertion^-Pro or pGreen II 0800-STP1^Deletion^-Pro, was used as a control. Data represent means ± standard deviation (*n* = 6). **e**, **f** Yeast one-hybrid assays of ZAT10-LIKE binding to the promoters of *STP1^Insertion^* (**e**) and *STP1^Deletion^* (**f**). The full-length CDS of *ZAT10-LIKE* was cloned into pGADT7 (AD) to generate the prey vector, pGADT7-ZAT10-LIKE. The promoters of *STP1^Insertion^* (**e**) and *STP1^Deletion^* (**f**) were cloned into pAbai to generate bait vectors, pAbai-STP1^Insertion^ (**e**) and pAbai-STP1^Deletion^ (**f**), respectively. The bait vector and the prey vector were introduced into Y1HGold yeast. A combination introducing the bait vector and the empty vector pGADT7 was used as a control. The transformants were cultured on SD/−Ura−Leu media with different concentrations of aureobasidin A (AbA). **g** Diagram of probes of EMSAs. The sequence in red is the 21-bp insertion. Sequences of Probe-STP1^Insertion^ and Probe-STP1^Deletion^ were derived from natural accessions. **h** EMSA assays of ZAT10-LIKE binding to the probes of *STP1^Insertion^* and *STP1^Deletion^*. + and − represent presence and absence, respectively. 5×, 10×, 20×, and 30× represent different competition multiples.

To determine the regulatory effect of ZAT10-LIKE or ZAT10 on the transcription of *STP1^Insertion^* and *STP1^Deletion^*, luciferase (LUC) assays were performed. We used CaMV35S promoter-driven ZAT10-LIKE and ZAT10 effectors (pGreen II 62-SK) and LUC reporters driven by the promoters of *STP1^Insertion^* and *STP1^Deletion^* (pGreen II 0800-LUC) to perform transient transactivation assays. Both effector and reporter vectors were co-transformed into *Agrobacterium* GV3101 cells and then the ratios of firefly LUC to *Renilla* luciferase (REN) were determined. The LUC to REN ratio obtained by co-transformation of ZAT10-LIKE effector with *STP1^Insertion^* was significantly higher than the control (Fig. 5a and c), while there was no marked difference in the ratios of LUC to REN obtained in co-transformation of ZAT10-LIKE effector with *STP1^Deletion^* and the control (Fig. 5b and d). However, there was neither a marked difference in the ratios of LUC to REN obtained between co-transformation of ZAT10 effector with *STP1^Insertion^* and the control nor co-transformation of ZAT10 effector with *STP1^Deletion^* and the control (Supplementary Data Fig. S5). Collectively, our results indicate that ZAT10-LIKE, but not ZAT10, can activate the expression of *STP1^Insertion^* but does not exert a regulatory effect on the *STP1^Deletion^* promoter.

In order to verify whether the different expression levels of *STP1^Insertion^* and *STP1^Deletion^* were caused by the differential binding capacity of ZAT10-LIKE to the *STP1* promoters, we conducted yeast one-hybrid assays. This confirmed that ZAT10-LIKE could specifically bind to the promoter of *STP1^Insertion^* but not to the promoter of *STP1^Deletion^* (Fig. 5e and f).

To further study the interactions between ZAT10-LIKE and the promoters of *STP1^Insertion^* and *STP1^Deletion^*, we performed electrophoretic mobility shift assays (EMSAs). We designed two 5'-fluorescein amidite (5'-FAM) -labeled probes, a 39-bp Probe-STP1^Insertion^ with the 21-bp insertion in the center, and an 18-bp Probe-STP1^Deletion^ without the 21-bp insertion (Fig. 5g). EMSAs indicated that ZAT10-LIKE can bind to Probe-STP1^Insertion^ but not to Probe-STP1^Deletion^ (Fig. 5h).

### *STP1* mutation exerts broad effects on gene expression and metabolite accumulation

**Figure 6:**
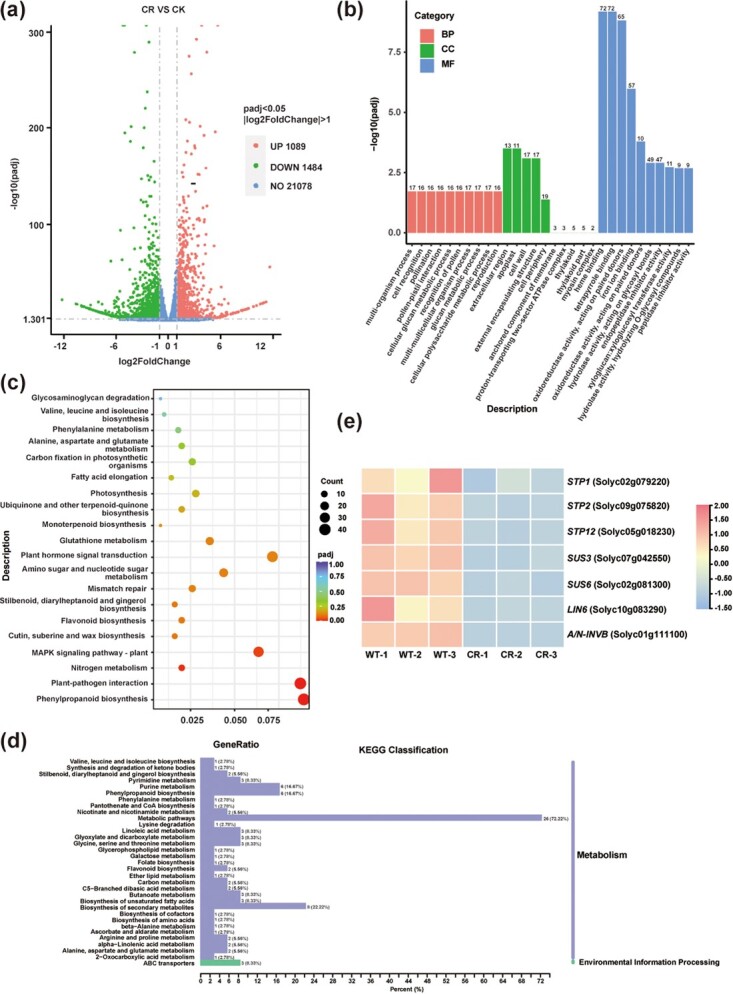
DEGs and metabolites in *STP1* knockout lines and WT. **a** Volcano map of DEGs. **b**, **c** Significantly enriched GO terms (**b**) and KEGG pathways (**c**) of DEGs. (**d**) KEGG pathways of differentially expressed metabolites. **e** Heat map of gene expression related to sugar metabolism from RNA-seq.

To study the effect of *STP1* mutation on whole-genome gene expression patterns, RNA-seq was performed in *STP1* knockout lines and WT. There were 2573 differentially expressed genes (DEGs), including 1089 upregulated genes and 1484 downregulated genes (Fig. 6a). GO (Gene Ontology) terms of DEGs included iron ion binding, oxidoreductase activity, tetrapyrrole binding, heme binding, extracellular region and apoplast (Fig. 6b). KEGG (Kyoto Encyclopedia of Genes and Genomes) pathways of DEGs included plant–pathogen interaction, nitrogen metabolism, phenylpropanoid biosynthesis, and MAPK signaling pathway (Fig. 6c).

We detected the content of primary metabolites between *STP1* knockout lines and WT, and a total of 592 primary metabolites were identified. Further analysis revealed that 106 differentially enriched metabolites (DEMs) were identified, of which 36 were significantly enriched metabolites. The annotation results for the DEMs using KEGG indicated that ~72.22% of DEMs were involved in metabolic pathways, 22.22% of DEMs were involved in biosynthesis of secondary metabolites, and 16.67% of DEMs were involved in phenylpropanoid biosynthesis (Fig. 6d). These results are consistent with those from RNA-seq. Biological processes are complex and integrative, and the combined analysis of multi-omics data is helpful for the study of phenotypes and regulatory mechanisms of biological processes [41]. Combining metabolite and transcriptome analysis, we found that 44 DEMs and 6 DEGs were involved in phenylpropanoid biosynthesis and 28 DEMs and 2 DEGs participated in carbon metabolism (Supplementary Data Fig. S6). The results of the combined analysis suggest that these DEMs may be regulated by DEGs. Overall, our results suggest that *STP1* mutation had large-scale effects on gene expression and metabolite accumulation.

More interestingly, the expression levels of genes related to sugar metabolism were significantly downregulated, such as *Sugar Transporter Protein 2* (*STP2*), *Sugar Transporter Protein 12* (*STP12*), *Sucrose Synthase 3* (*SUS3*), *Sucrose Synthase 6* (*SUS6*), *Invertase 6* (*LIN6*), and *Neutral/alkaline Invertase* (*A/N-INVB*) (Fig. 6e). Sucrose synthase, invertase and neutral/alkaline invertase are related to conversion of sucrose to glucose and fructose [42]. These results indicate that the decreased content of glucose and fructose in *STP1* knockout lines is probably due to decreased cleavage of sucrose.

### *STP1* InDel_21 was selected during tomato improvement

**Figure 7:**
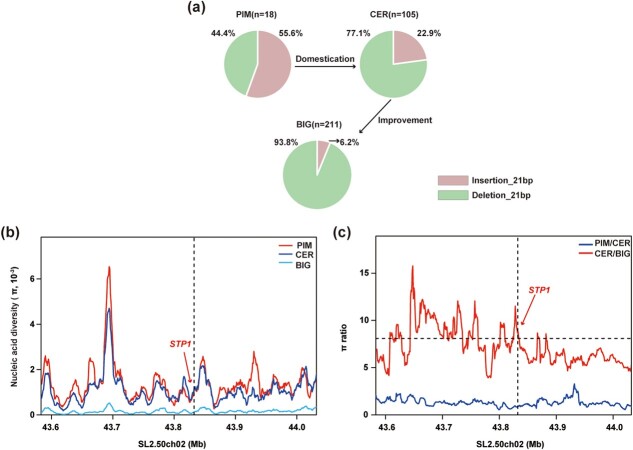
Analysis of molecular diversity of *STP1* during domestication and improvement. **a** Frequency of InDel_21bp allele in tomato subpopulations. *n* represents the number of accessions. **b** Distribution of nucleotide diversity (π) of PIM (red line), CER (navy blue line), and BIG (light blue line) within the 43.6–44 Mb region on chromosome 2. Red arrows, *STP1*. **c** Ratios of nucleotide diversity (π) between PIM and CER or between CER and BIG accessions on chromosome 2. Red arrows, *STP1*.

Since InDel_21 appeared to be the causal variation responsible for the differences in *STP1* expression and SSC content, we investigated InDel_21 variants in a panel of 334 accessions, containing 18 PIM, 105 CER, and 211 BIG accessions. A total of 55.6% (10 of 18) of the PIM accessions carried Insertion_21, 22.9% (24 of 105) of the CER accessions carried Insertion_21 and 6.2% (13 of 211) of the BIG accessions carried Insertion_21 (Fig. 7a). Nucleotide diversity (π) in PIM, CER, and BIG accessions was measured (Fig. 7b) and the π ratios between groups were calculated to detect putative domestication (πPIM/πCER = 0.92) and improvement (πCER/πBIG = 8.50) sweeps (Fig. 7c). Our data provide proof that *STP1* was selected during tomato improvement.

## Discussion

Plant domestication, which can create a new plant form to meet human needs, is the genetic modification of wild species [43]. Food crops typically have enhanced determinate growth or increased apical dominance, more robust growth habit, larger fruits or grains, and a loss of natural seed dispersal for easy harvest by humans compared with their progenitors [43]. Tomato domestication has greatly increased fruit yield [44]. However, there was a negative relationship between SSC and fresh weight in fruits so that it was difficult to transfer high-SSC traits from wild species into cultivated varieties [44]. Although tomato varieties with improved traits have been generated successfully [45, 46], the efficiency of genetic improvement of SSC is still relatively limited [47].

The sequencing of the tomato genome [10] and resequencing of hundreds of natural accessions [11, 48] has accelerated the process of gene/allele discovery by GWAS, QTL sequencing and mapping-by-sequencing (MBS) [1]. Compared with traditional genetic populations, including NILs and RILs, GWAS saves time in building populations and provides richer allelic diversity. mGWAS has been widely used to study the evolution of tomato fruit quality [49–51] and a large number of genes related to tomato quality have been identified through mGWAS. Functional and evolutionary verification of candidate genes has, however, rarely been carried out, which hampers the use of natural variation to improve tomato flavor quality. Here we used a GWAS for SSC with 481 tomato accessions. Besides the previously reported locus, *LIN5* [6], we also found other QTLs on chromosomes 2, 5, 6, and 8 (Fig. 1a), and further analyzed the candidate genes on the four chromosomes (Supplementary Data Tables S3 and S6–S8). Using significant SNPs, sequence amplification, and gene annotation, we identified a candidate gene on chromosome 2, *STP1*, attributed to the variation of SSC. This locus has also been mentioned in previous studies by GWAS on glucose [51] and SSC [22]. However, its function and natural variation have not been identified. To further test whether *STP1* plays a key regulatory role in SSC, 13 accessions with high-SSC phenotypes and 8 accessions with low-SSC phenotypes were selected randomly and the expression of *STP1* was quantified in the red-ripe fruits using qRT–PCR. Our data showed that the expression of *STP1* in the high-SSC accessions was observably higher than that in the low-SSC accessions (Fig. 1g). We confirmed a sequence variation of a 21-bp InDel at 1124 bp upstream of the start codon in the *STP1* promoter. We characterized the 21-bp InDel variation in 334 tomato accessions with the cleaved amplified polymorphic sequence (CAPS)marker (Fig. 2a). *STP1* can be classified into two different alleles, *STP1^Insertion^* and *STP1^Deletion^* (Fig. 2b). The accessions with the 21-bp insertion mainly belong to PIM and CER, and the accessions without the 21-bp insertion mainly fall within BIG (Fig. 2b). Through phenotypic statistics, we found SSC of *STP1^Insertion^* accessions was significantly higher than that of *STP1^Deletion^* accessions (Fig. 2c). GUS staining showed stronger transcription activity by the promoter of *STP1^Insertion^* than by the *STP1^Deletion^* promoter (Fig. 2d and e). These results demonstrate that the 21-bp InDel does regulate the expression of *STP1*.

STP usually has glucose and fructose transport activity [27, 52, 53], and a related protein, MdSTP13a, also has sucrose transport activity [54]. Previous studies have shown that RNAi-mediated knockdown of *LeHT* genes led to a 55% decrease in fruit hexose accumulation [27] and heterologous expression of *LeHT1* in yeast can rescue a hexose transport-impaired mutant [27]. CRISPR/Cas9 technology was used to construct *STP1* knockout lines in TS-23 with high SSC, and the SSC decreased significantly in three knockout lines (Fig. 3b). Furthermore, the contents of glucose, fructose, and sucrose all significantly decreased, while the content of malic acid and citric acid was not altered (Fig. 3c–e; Supplementary Data Fig. S2). These data indicate that *STP1* positively regulates the SSC by regulating the contents of glucose, fructose, and sucrose in tomato fruits. To explore the potential mechanism underlying the altered sugar concentration, we further analyzed RNA-seq data of the red-ripe fruits in *STP1* knockout lines and WT. As expected, gene expression related to sugar metabolism was significantly downregulated, including *SUS3* (*Sucrose Synthase*), *SUS6* (*Sucrose Synthase*), *LIN6* (*Invertase 6*), and *A/N-INVB* (*Neutral/alkaline Invertase*), which are involved in sucrose dissociation (Fig. 6e). These findings further support the suggestion that sucrose cleavage decreased in *STP1* knockout lines, resulting in a decrease in glucose and fructose content.

In order to study the regulation by the 21-bp InDel in the promoter of the gene expression of *STP1*, we performed a yeast one-hybrid screening and identified a transcription factor, ZAT10-LIKE. We showed that ZAT10-LIKE can bind to the promoter of *STP1^Insertion^* but not to the promoter of *STP1^Deletion^* through LUC assays, EMSAs, and yeast one-hybrid assays (Fig. 5). Conserved domain analysis of ZAT10-LIKE revealed that it has a typical EAR motif at its C-terminus (Supplementary Data Fig. S7), which reportedly inhibits the transcription of target genes [55, 56]. However, some EAR domain-containing ZFP TFs may also activate other target genes [57, 58], and we speculate that other domains in ZAT10-LIKE may affect its transcriptional activity. ZFP TFs were involved in plant development and stress responses [29, 30], especially in salt stress [33, 59, 60], but little is known about their role in the regulation of metabolism. Our study demonstrates a novel function of ZFP TFs in regulating sugar transporters, but a detailed function of ZAT10-LIKE in regulating SSC needs to be further identified.

Finally, we investigated the InDel_21 variants among 334 accessions (Fig. 7a). We measured the level of nucleotide diversity (π) in PIM, CER, and BIG accessions and calculated the ratios of π between PIM and CER and between CER and BIG, which were suggestive of an improvement event (Fig. 7b and c). *STP1* was observed in an improvement sweep, which was consistent with the previous report [11].

In summary, we identified a genetic locus, *STP1*, which is required for increased SSC of red-ripe fruits in tomato. During tomato improvement, the removal of InDel_21 in the *STP1* promoter led to a decrease in fruit SSC. This research can help uncover the genetic basis and natural variation of primary metabolism. Our data indicate that *STP1* might have been a critical factor for high SSC in undomesticated plants and its restoration and/or modification in crops may improve SSC. We have also discovered a new role for ZFP TFs in regulating sugar transporters.

## Materials and methods

### Tomato accessions and genome-wide association study

Four hundred and eighty-one tomato accessions, including 6 *Solanum cheesmaniae* (wild), 22 *S. pimpinellifolium* (PIM), 118 *S. lycopersicum* var. *cerasiforme* (CER), 275 *S. lycopersicum* (BIG) and 60 Guangxi accessions collected by our laboratory (Supplementary Data Table S1), were used to perform GWAS conducted in a greenhouse at Wuhan Agricultural Academy of Sciences, China, in Spring 2016. A total of 4 540 171 SNPs (minor allele frequency >5% and missing rate <10%) for 481 accessions from the previously study [48] were used for GWAS. Beagle software was chosen to estimate missing genotypes [61] and the efficient mixed-model association expedited (EMMAX) algorithm was chosen to perform the association analysis [62]. The effective number of independent SNPs was calculated by GEC software [63] and a total of 1 007 010 independent SNPs were obtained. The significance *P* threshold was determined by Bonferroni correction as *P* = α/*n* (*n* = 1 007 010). The suggestive and significant thresholds were imputed to be 9.930388 × 10^−7^ (α = 1) and 4.965194 × 10^−8^ (α = 0.05), respectively. The physical locations of the SNPs were based on the tomato reference genome sequence version SL2.50 (http://solgenomics.net). The results of GWAS for specific candidate segments were visualized with LocusZoom software [64]. The linkage segment of each significant locus was calculated using LDBlockShow software [65], and the linkage segment harboring significant SNPs was used as a candidate region.

### RNA extraction and gene expression analysis

Total RNA was extracted using the Trizol reagent (Invitrogen, USA) and further reverse transcribed into cDNA using a HiScript II 1st Strand cDNA Synthesis Kit (Vazyme, China). The relative transcript levels of specific genes were quantified using qRT–PCR. The expression level of the *Actin* gene (*Solyc11g005330*) was used as reference [39]. Related primer sequences can be found in Supplementary Data Table S2.

### Gene cloning, vector construction, and transformation

*STP1* knockout lines were generated using pTX vector, a CRISPR/Cas9 binary vector. TS-23 with high SSC was transformed using *Agrobacterium* (strain GV3101)-mediated transformation [66]. A total of three homozygous knockout lines were chosen for further analysis.

### DNA sequencing and CAPS markers for the 21-bp InDel

To detect variation in the *STP1* gene, specific primers were designed to perform PCR amplification of a 2-kb *STP1* promoter region and full-length genomic DNA from 10 accessions with extremely high SSC and 10 with extremely low SSC accessions, and the PCR products were confirmed by sequencing. *STP1^Insertion^* and *STP1^Deletion^* accessions were genotyped using CAPS markers. Specific primers were designed to perform PCR amplification of *STP1* promoter sequences containing the 21-bp InDel. The PCR products were subsequently digested with *Bsr*I (New England Biolabs, USA) for 3 hours at 65°C and then incubated for 20 minutes at 85°C in a reaction containing 10 μl PCR products, 7.6 μl double-distilled water, 2 μl 10 × NEBuffer 3.1, and 0.4 μl *Bsr*I*.* The PCR products of *STP1^Insertion^* accessions were designed to generate bands ~380 bp in length, while the PCR products of *STP1^Deletion^* accessions could not be digested by BsrI.

### GUS staining

The promoters of *STP1* were amplified from TS-23 (*STP1^Insertion^* accession) and TS-9 (*STP1^Deletion^* accession), and then inserted into the pHELLSGATE8 vector without the CaMV35S promoter to generate GUS reporter constructs, proSTP1^Insertion^::GUS and proSTP1^Deletion^::GUS. These two reporter constructs were injected into tobacco leaves and the leaves were collected 48 hours after infiltration. One half of each leaf was used to determine the expression of the *GUS* gene and the other half was stained. *GUS* expression was quantified using qRT–PCR. For GUS staining, the tobacco leaves were incubated with staining buffer for 12 hours at 37°C in the dark and faded with 70% (v/v) ethanol.

### Phenotyping

SSC was quantified as Brix in total fruit juice from red-ripe fruits using a digital refractometer (PAL-BX|ACID 3). Glucose, fructose, sucrose, malate, and citric acid were measured by gas chromatography (GC). The red-ripe fruits were ground with liquid nitrogen and further extracted with 80% methanol. The extracted samples were concentrated *in vacuo* and then derivatized with hydroxylamine hydrochloride, hexamethyldisilane (HMDS, Sigma), and trimethylchlorosilane (TMCS, Sigma). Derivatized supernatants were added to a 2-ml automatic sample injection bottle for GC-FID analysis.

### Subcellular localization

The full-length coding sequences (CDSs) of *ZAT10-LIKE* and *ZAT10* without the termination codons were amplified from the cDNA of ‘Ailsa Craig’ (AC) and cloned into 101LYFP containing the CaMV35S promoter [67]. *Agrobacterium* strain GV3101 containing CaMV35S: ZAT10-LIKE-YFP or CaMV35S: ZAT10-YFP and the cell nucleus marker CaMV35S: StERF3-RFP [68] was infiltrated into tobacco leaves. After 48 hours of incubation, the image of yellow fluorescent protein (YFP) and red fluorescent protein (RFP) fluorescence was captured by confocal laser scanning microscopy (Leica SP8SP8, Germany).

### Yeast one-hybrid assay

The 21-bp insertion sequence in the *STP1* promoter was taken as the center, and 9-bp fragments were added to the left and right sides, yielding an 18-bp *STP1^Deletion^* fragment and a 39-bp *STP1^Insertion^* fragment. The 18- or 39-bp fragments were used as repeats and fragments with five repeats in tandem were artificially synthesized. The synthesized fragments were connected to pAbai vectors and then used for yeast one-hybrid screening. The full-length CDS of *ZAT10-LIKE* was amplified from the cDNA of AC and cloned into a pGADT7 vector. Yeast strains were chosen and dissolved in 100 μl double-distilled water. PCR amplification was carried out with universal primers. PCR products were confirmed by sequencing. Heinz 1706 (LA3530) was used for reference.

Both the pGADT7 prey vector and the bait vector were introduced into Y1HGold yeast (Clontech, USA) and grown on SD/−Ura−Leu media. After 3 days of culture, the positive yeast strains were picked and dissolved in double-distilled water, and a suspension was spotted onto SD/−Ura−Leu media with different concentrations of aureobasidin A (AbA).

### Electrophoretic mobility shift assay

We first amplified the full-length CDS of *ZAT10-LIKE* from the cDNA of AC, and transferred it into *Eco*RI and *Xho*I-digested PGEX-4 T-1 (GST) vector by homologous recombination. Protein with a GST tag was expressed in *Escherichia coli* BL21 (DE3) cells and then cultured to an OD_600_ of 0.6. Protein was further induced with isopropyl-β-d-thiogalactopyranoside (IPTG) for 20 hours at 16°C and purified. The *STP1^Insertion^* and *STP1^Deletion^* probes were labeled with FAM and are shown in Fig. 5g. FAM was attached to the 5′ end of the sense oligonucleotides to label the probes. The double-stranded probes were formed by annealing the single-stranded probes and then dissolved in double-distilled water to 10 μM. For competitor probes, the double-stranded probe was used directly in 100 μM after annealing. ZAT10-LIKE protein was incubated with double-stranded probes with a 20-μl reaction mixture under dark conditions. After 30 minutes at 4°C, the 6% non-denaturing polyacrylamide gels were used to separate the mixture and the image was captured using a multifunctional imaging analysis system (ProteinSample) after 1 hour of migration.

### Dual luciferase transactivation assay

The *STP1* promoters (−1 to 1187 bp) were amplified from TS-23 and TS-9 and then cloned into the pGreen II 0800-LUC vector to generate reporter constructs. The full-length CDSs of *ZAT10-LIKE* and *ZAT10* were cloned into the pGreen II 62-SK vector to generate effector constructs. *Agrobacterium* strains containing a reporter construct and an effector construct were infiltrated into tobacco leaves. After 3 days of incubation, firefly luciferase (LUC) and *Renilla* luciferase (REN) activity was determined and the ratios of LUC to REN were calculated to indicate transactivation activity.

### RNA-seq

Samples from *STP1* knockout lines and WT were collected with three biological replicates and each replicate contained 18 red-ripe fruits from three plants. RNA was extracted and RNA-seq was performed using a HiSeq-PE150 sequencing system. We used the average FPKM (expected number of fragments per kilobase of transcript sequence per million base pairs sequenced) value as a measure of gene expression [69]. A gene yielding >2-fold expression with *P* ≤ .05 was classified as a DEG.

### Metabolome profiling

Samples of red-ripe fruits were collected from *STP1* knockout lines and WT to detect primary metabolites (Wuhan, China; http://www.metware.cn/). Metabolome profiling was carried out according to the method of Liu *et al*. [70].

### Molecular diversity analysis

We used the nucleotide diversity (π) ratio to identify selective sweeps for the molecular diversity analysis of *STP1* in tomato. VCFtools was used to measure the levels of π in PIM, CER, and BIG accessions in 10-kb windows, with a step size of 1 kb [71]. The π ratios (πPIM/πCER, and πCER/πBIG) were calculated. Windows with the top 5% of ratios were selected for further analysis (3.02 and 8.09 for domestication and improvement, respectively).

## Acknowledgements

This work was supported by grants from the National Key Research & Development Plan (2021YFD1200201; 2022YFD1200502), the National Natural Science Foundation of China (31972426; 31991182; 32060685), the Wuhan Biological Breeding Major Project (2022021302024852), the International Cooperation Promotion Plan of Shihezi University (GJHZ202104), the Key Project of Hubei Hongshan Laboratory (2021hszd007), the Hubei Key Research & Development Plan (2022BBA0062; 2022BBA0066), and the Fundamental Research Funds for the Central Universities (2662022YLPY001).

## Author contributions

Y.Z. designed and supervised the project. Y.W. did most of the experiments and wrote the manuscript. C.S. performed the GWAS data analysis. P.G., F.L., L.Z., Y.W., J.T., X.Z., H.D., W.G., and F.W. performed some of the experiments. W.X., D.G., Y.Z., and Z.Y. advised on experiments and revised the manuscript.

## Conflict of interest statement

All authors declare no conflicts of interest.

## Data availability

All data supporting the results of this study can be obtained in this article or in the supplementary materials.

## Supplementary data

Supplementary data is available at *Horticulture Research* online.

## Supplementary Material

Web_Material_uhad009
