# Supplementary material for: A 21-bp InDel in the promoter of STP1 selected during tomato improvement accounts for soluble solid content in fruits
Source: Hortic Res. 2023 Feb 10;10(3):uhad009. doi: 10.1093/hr/uhad009 (PMC10028405; doi:10.1093/hr/uhad009)
Supplement: Web_Material_uhad009 [file web_material_uhad009.zip › Supplemental Figures.docx]

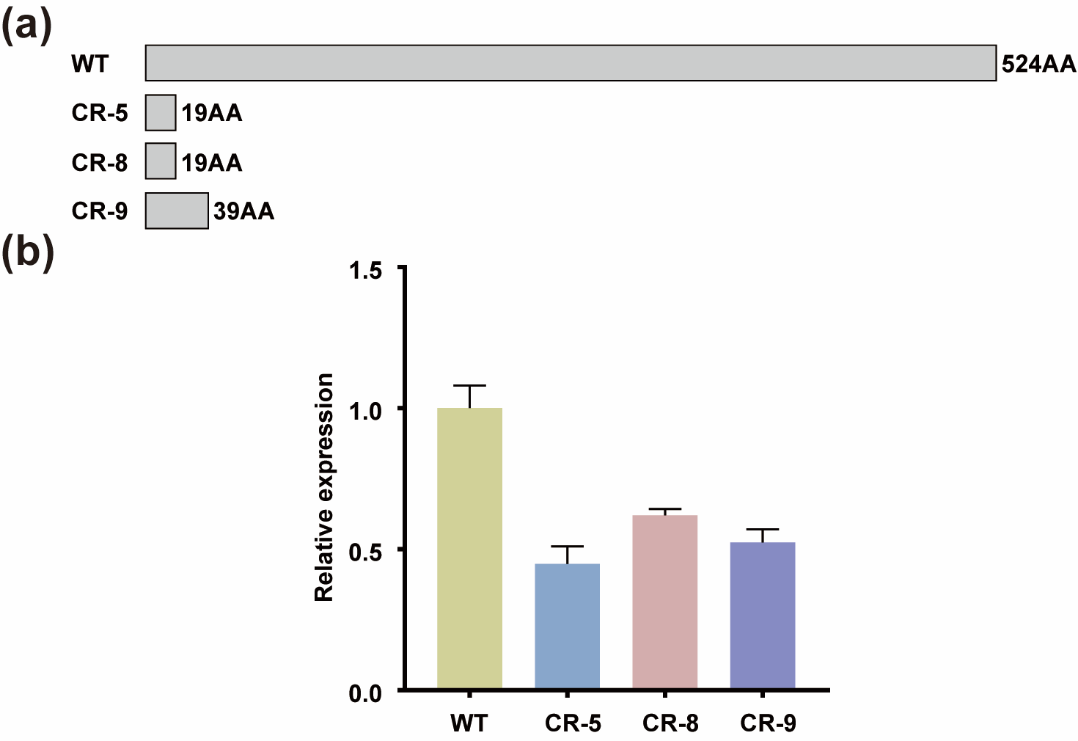


**Fig. S1.** Schematic diagram of the truncated amino acids and reduced *STP1* expression in *STP1* knockout lines relative to WT. **(a)** Schematic diagram of the truncated amino acids in *STP1* knockout lines relative to WT. **(b)** Reduced *STP1* expression in the red-ripe fruits of *STP1* knockout lines and WT. Values are represented as means ± SD (n = 3).


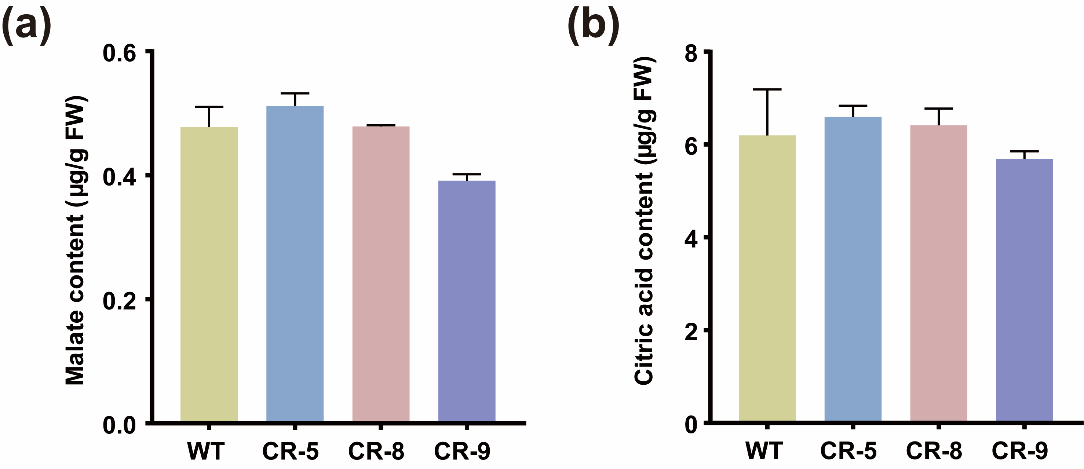


**Fig. S2.** The content of malate and citric acid in *STP1* knockout lines and WT. **(a)** Malate determination in the red-ripe fruits of three knockout lines and WT. **(b)** Citric acid determination in the red-ripe fruits of three knockout lines and WT. Values are represented as means ± SD (n = 3).


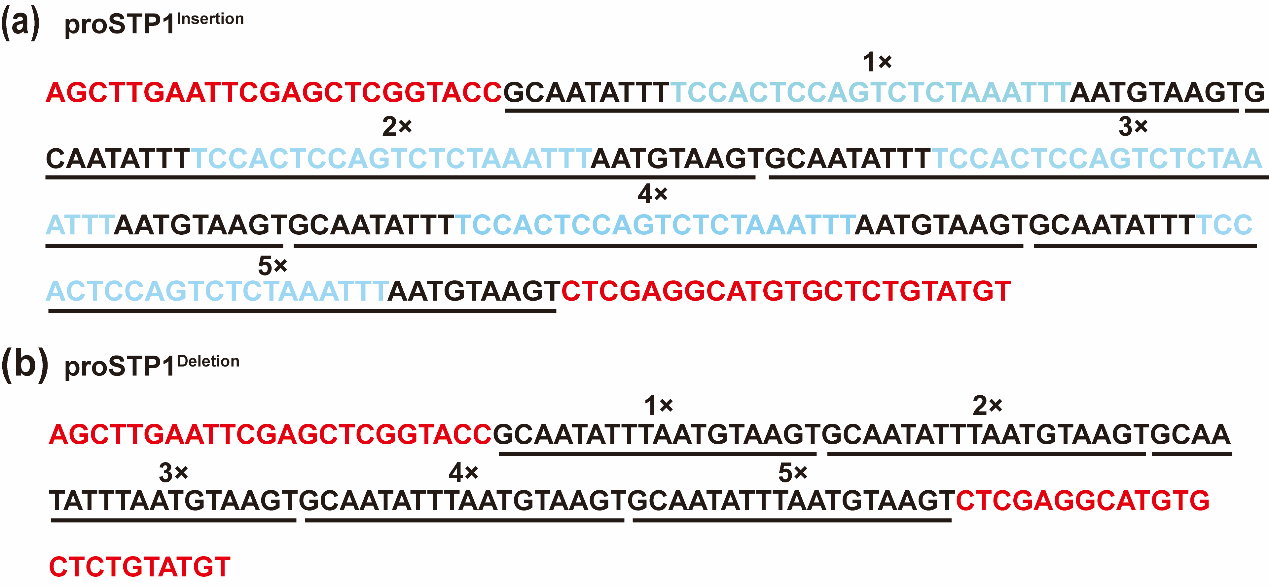


**Fig****. S3.** Bait sequences with 5-fold tandem repeat (for yeast one-hybrid screening). **(a)** Bait sequence of proSTP1^Insertion^. Sequence in red indicates the homologous recombination linker of the pAbai vector and sequence in blue indicates 21-bp insertion. 1×, 2×, 3×, 4× and 5× represent the multiples of tandem repeat, respectively. **(b)** Bait sequence of proSTP1^Deletion^. Sequence in red indicates the homologous recombination linker of the pAbai vector. 1×, 2×, 3×, 4× and 5× represent the multiples of tandem repeat, respectively. Each black-line segment represents a tandem repeat.


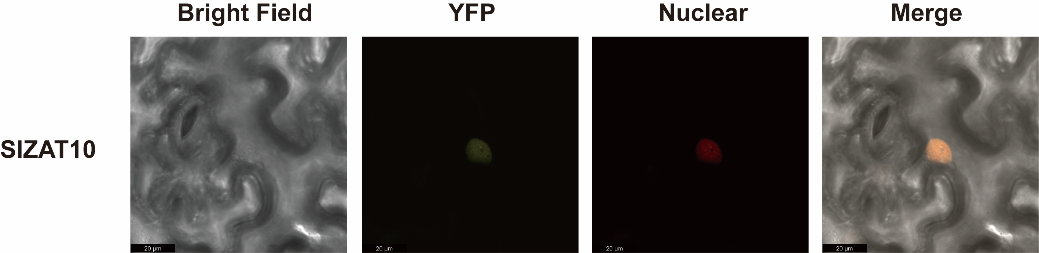


**Fig. S4.** Subcellular localization of ZAT10-YFP fusion protein. Tobacco leaves were transiently transformed with the fusion construct (ZAT10-YFP) and a nuclear marker (StERF3-RFP). Bright-field, yellow fluorescent protein, red fluorescent protein and merged images are shown.


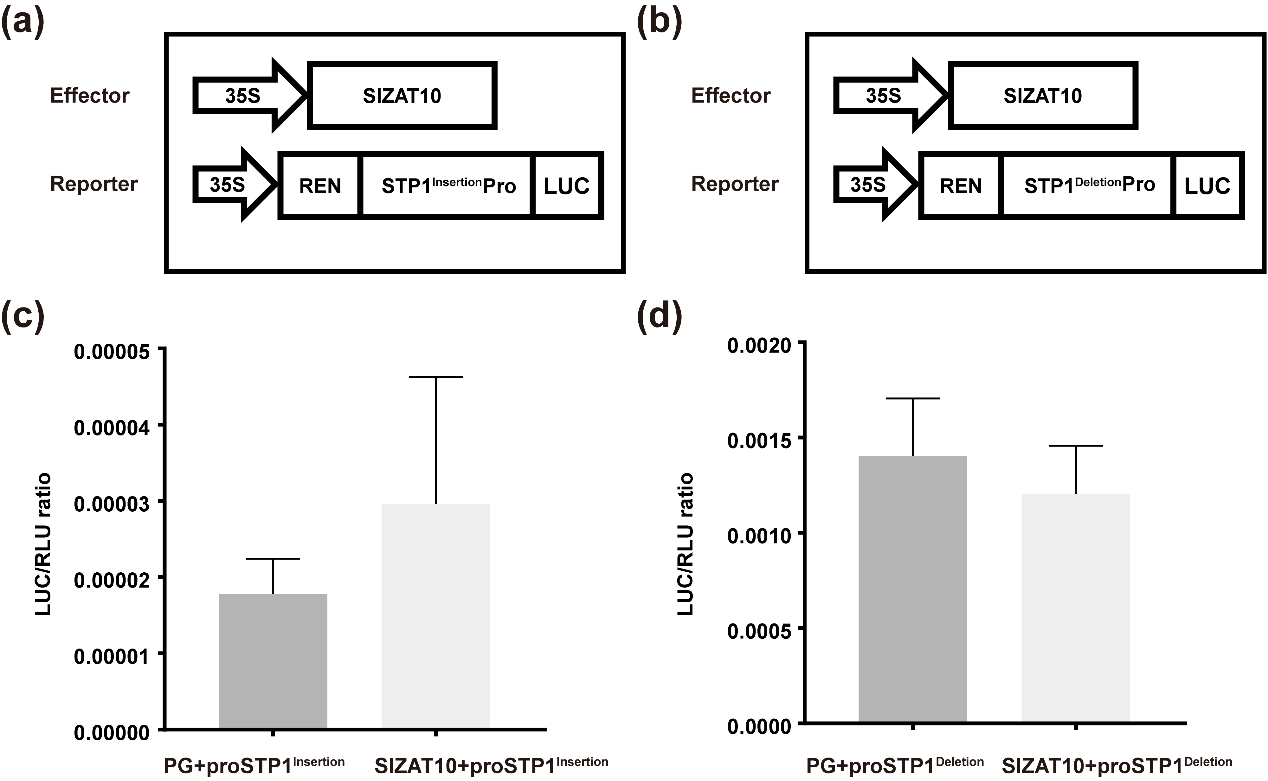


**Fig. S5.** Differential binding capacity of ZAT10 to the promoters of *STP1^Insertion^* and *STP1^Deletion^*. (**a** and **b**) Schematic representation of constructs used for the dual luciferase assays. The full-length CDS of ZAT10 was cloned into pGreen II 62-SK to generate the effector construct, pGreen II 62-Sk-ZAT10. The promoter fragments of *STP1* (-1 to -1187), amplified from TS-23 and TS-9 respectively, were fused to pGreen II 0800-LUC to create the reporter constructs, pGreen II 0800-STP1^Insertion^-Pro (**a**) and pGreen II 0800-STP1^Deletion^-Pro (**b**). (**c** and **d**) Relative LUC/REN ratios were used to evaluate the promoter activity of *STP1^Insertion^* (**c**) and *STP1^Deletion^* (**d**) in the presence of ZAT10. PG, the pGreenII 62-SK empty vector with pGreen II 0800- STP1^Insertion^-Pro or pGreen II 0800- STP1^Deletion^-Pro, was used as a control. LUC, firefly luciferase activity; REN, renillia luciferase activity. Data are shown as means ± SD (n = 6).


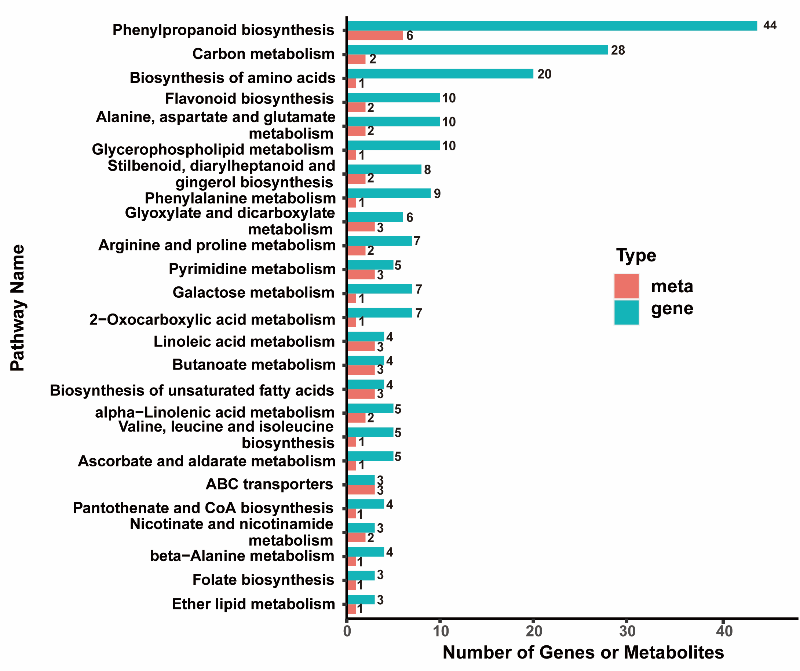


**Fig. S6.** KEGG pathways for combined transcriptome and metabolome analysis. The x-axis represents the number of differential metabolites and differential genes enriched in the pathway, the y-axis represents the name of the KEGG pathway, and the red and green bars represent the metabolome and transcriptome, respectively.


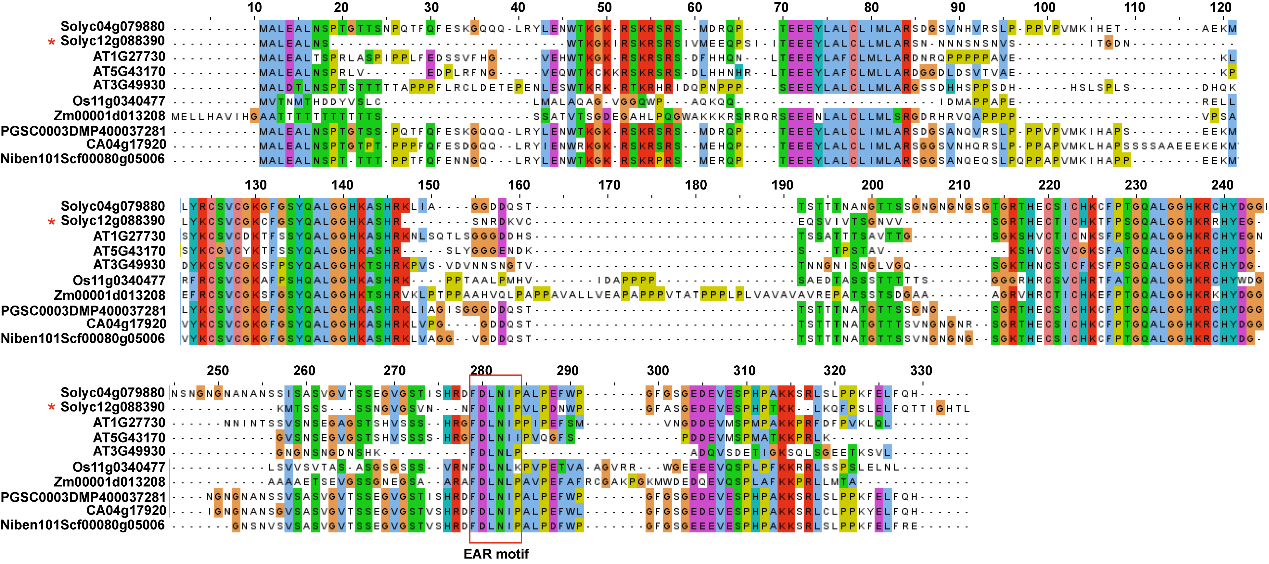


**Fig. S7.** EAR domain analysis of ZAT10-LIKE (Solyc12g088390) and its homologous genes. The gene marked with a red asterisk is *ZAT10-LIKE.* Putative EAR motifs are boxed in red.
